# NOR-1/NR4A3 regulates the cellular inhibitor of apoptosis 2 (cIAP2) in vascular cells: role in the survival response to hypoxic stress

**DOI:** 10.1038/srep34056

**Published:** 2016-09-22

**Authors:** Judith Alonso, María Galán, Ingrid Martí-Pàmies, José María Romero, Mercedes Camacho, Cristina Rodríguez, José Martínez-González

**Affiliations:** 1 Centro de Investigación Cardiovascular (CSIC-ICCC), Instituto de Investigación Biomédica Sant Pau (IIB-Sant Pau), c/Sant Antoni Maria Claret 167, 08025 Barcelona, Spain; 2 Laboratorio de Angiología, Biología Vascular e Inflamación y Servicio de Cirugía Vascular, IIB-Sant Pau, c/Sant Antoni Maria Claret 167, 08025 Barcelona, Spain

## Abstract

Vascular cell survival is compromised under pathological conditions such as abdominal aortic aneurysm (AAA). We have previously shown that the nuclear receptor NOR-1 is involved in the survival response of vascular cells to hypoxia. Here, we identify the anti-apoptotic protein cIAP2 as a downstream effector of NOR-1. NOR-1 and cIAP2 were up-regulated in human AAA samples, colocalizing in vascular smooth muscle cells (VSMC). While NOR-1 silencing reduced cIAP2 expression in vascular cells, lentiviral over-expression of this receptor increased cIAP2 mRNA and protein levels. The transcriptional regulation of the human cIAP2 promoter was analyzed in cells over-expressing NOR-1 by luciferase reporter assays, electrophoretic mobility shift analysis and chromatin immunoprecipitation, identifying a NGFI-B site (NBRE-358/-351) essential for NOR-1 responsiveness. NOR-1 and cIAP2 were up-regulated by hypoxia and by a hypoxia mimetic showing a similar time-dependent pattern. Deletion and site-directed mutagenesis studies show that NOR-1 mediates the hypoxia-induced cIAP2 expression. While NOR-1 over-expression up-regulated cIAP2 and limited VSMC apoptosis induced by hypoxic stress, cIAP2 silencing partially prevented this NOR-1 pro-survival effect. These results indicate that cIAP2 is a target of NOR-1, and suggest that this anti-apoptotic protein is involved in the survival response to hypoxic stress mediated by NOR-1 in vascular cells.

Vascular remodelling enables the healing and adaptation of blood vessels to mechanical injury or hemodynamic changes, and underlies pathogenic processes such as atherosclerosis, restenosis and abdominal aortic aneurysm (AAA)^1^,^2^. Apoptosis of vascular smooth muscle cells (VSMC) is critical in vascular remodelling, and it is significantly increased in vascular pathologies such as AAA^1^,^3^. Indeed, AAA is a complex age-related degenerative disease with high mortality rate, characterized by the degradation of vascular extracellular matrix (ECM) components and by the loss of the vascular cellularity due to increased VSMC apoptosis^1^.

Apoptosis can be initiated through two main pathways (the extrinsic and the intrinsic) and involves an amplifying proteolytic cascade which leads to the consummation of apoptosis^4^. The members of the inhibitor of apoptosis (IAP) family are critical proteins regulating apoptosis^5^. IAPs are structurally related proteins that promote pro-survival signalling pathways and prevent the activation of the effector phase of apoptosis by interfering caspase activity. All IAPs contain the signature baculoviral IAP repeat (BIR), some of them have carboxy-terminal RING domains that function as ubiquitin ligases^5^,^6^,^7^, and cIAP1 and cIAP2 also possess a caspase recruitment domain (CARD) and an ubiquitin-associated domain (UBA)^5^,^8^. cIAP2 (also known as HIAP1 or BIRC3) is a potent inhibitor of apoptotic death that, in contrast to other members of the IAP family, is transcriptionally inducible by a number of triggers in different cell types including vascular cells^9^,^10^,^11^, and is up-regulated in human tissues such as atherosclerotic plaques^12^,^13^.

We and others have recently involved neuron-derived orphan receptor 1 (NOR-1) in vascular remodelling and coronary artery disease (CAD)^14^,^15^,^16^,^17^,^18^. NOR-1 (NR4A3) is a member of the NR4A subfamily of nuclear receptors^19^,^20^,^21^. These nuclear receptors seem to be constitutively active, ligand-independent transcription factors^22^, expressed at low levels in resting vascular cells but quickly induced by extracellular cues, acting as early-response genes^19^,^20^,^21^. NOR-1, that is up-regulated by a variety of stimuli including growth factors and molecules with mitogen-like activity such as lipoproteins or thrombin^14^,^23^,^24^,^25^,^26^,^27^, regulates vascular cell spreading, migration and proliferation^14^,^17^,^26^,^27^,^28^,^29^. Furthermore, we have shown that NOR-1 is implicated in the survival response of endothelial cells to hypoxia^11^. Current information on NOR-1 target genes in the vasculature, however, is very limited. By knockdown experiments in endothelial cells we early suggested that cIAP2 could be a target of NOR-1^11^. In the present study we show that cIAP2 is a direct target of NOR-1, and analyze the role of this anti-apoptotic protein in the pro-survival effects of NOR-1 in response to hypoxic stress.

## Results

### The expression of NOR-1 and cIAP2 is increased in AAA tissues

**Figure 1:**
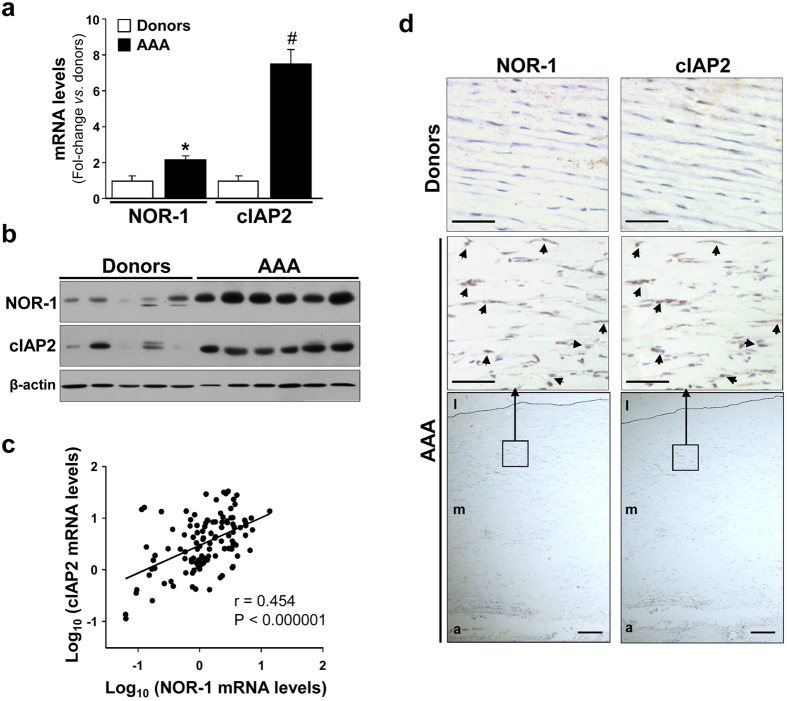
NOR-1 and cIAP2 are over-expressed in human AAA tissues. (**a**) NOR-1 and cIAP2 mRNA levels determined by real-time PCR in human AAA samples (black bars; n = 96) and human healthy aortic wall (Donors; white bars; n = 16). Data are expressed as mean ± s.e.m. *P < 0.01 *vs*. Donors; ^#^P < 0.0001 *vs*. Donors. β-actin was used as endogenous control. (**b**) Representative western blot showing NOR-1 and cIAP2 protein levels in these tissues. Levels of β-actin were used as a loading control. (**c**) Positive statistical correlation between mRNA levels of NOR-1 and cIAP2 in patients and donors. The Spearman Rank Order Correlation after logarithmic transformation of data was applied (n = 112; r = 0.454; P < 0.000001). (**d**) Representative high magnification images showing the immunohistochemical analysis of NOR-1, and cIAP2 in consecutive sections from human healthy aortic wall samples (Donors; upper panels) and human AAA (middle panels). Large lower panels show low magnification images from AAA immunostaining. The position of the corresponding high power views squared. Bar = 50 μm (in high magnification images); Bar = 200 μm (in low magnification images). A: adventitia, m: media, and l: lumen.

We analyzed the expression of NOR-1 and cIAP2 in human AAA tissues, samples in which vascular cells are exposed to conditions that compromise their survival, and in aortas from healthy donors. The expression of both genes was significantly enhanced in AAA samples as we showed by real-time PCR and Western blot (Fig. 1a,b). Interestingly, in these tissues NOR-1 mRNA levels significantly correlated with those of cIAP2 (n = 112; r = 0.454; P < 0.000001; Fig. 1c). Immunohistochemistry analysis of consecutive AAA sections evidenced that NOR-1 colocalizes with cIAP2 in VSMC in the tunica media (Fig. 1d).

### NOR-1 is necessary for cIAP2 expression in human vascular cells

**Figure 2:**
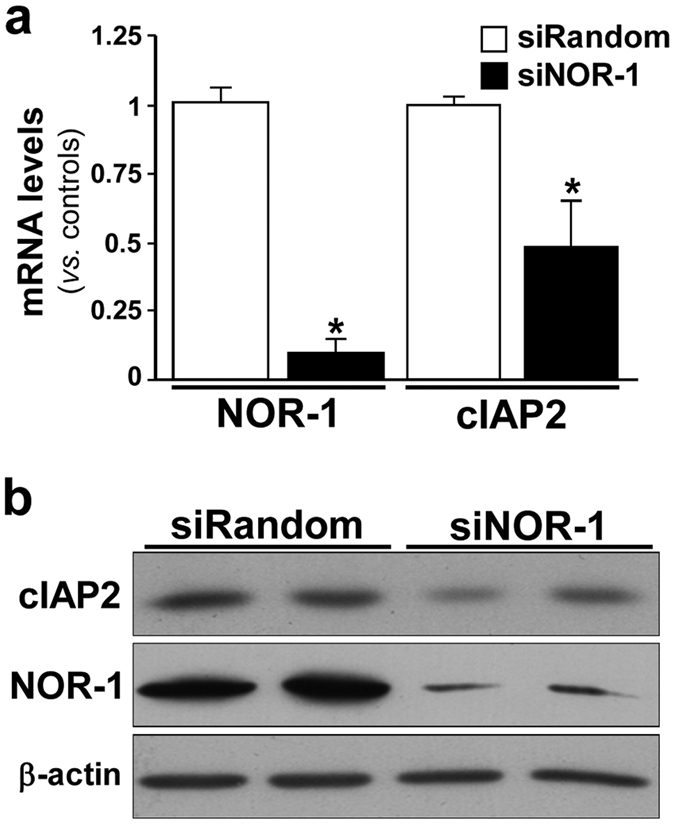
NOR-1 knockdown down-regulates cIAP2 expression in VSMC. Human VSMC were transfected with a siRNA against NOR-1 (siNOR-1, ID s15541; black bars) or a random siRNA (siRandom; white bars). (**a**) The expression of NOR-1 and that of cIAP2 in these samples was analyzed by real-time PCR (n = 6). *P < 0.0001 *vs.* siRandom. (**b**) The down-regulation of cIAP2 by siNOR-1 was also confirmed by western blot. Levels of β-actin were used as a loading control.

**Figure 3:**
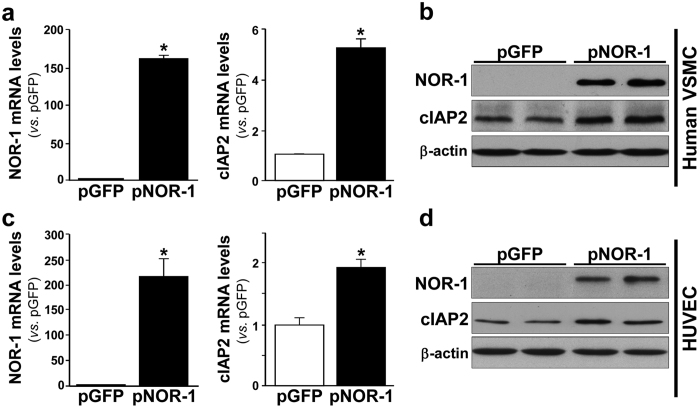
NOR-1 over-expression increases cIAP2 expression in vascular cells. Analysis of NOR-1 and cIAP2 mRNA (**a**,**c**) and protein levels (**b**,**d**) in human VSMC (**a**,**b**) and endothelial cells (**c**,**d**) transfected to over-express NOR-1 (pNOR-1) or GFP (pGFP; control cells) (n = 5). *P < 0.0001 *vs*. control cells. Levels of β-actin are shown as a loading control in western blot analysis.

We have previously shown that NOR-1 silencing decreased cIAP2 mRNA levels in human endothelial cells^11^. Here we show that, in human VSMC, NOR-1 knockdown significantly decreased cIAP2 mRNA and protein levels (Fig. 2a,b). The inhibitory effect of NOR-1 silencing on cIAP2 expression was confirmed using a pool of siRNAs against NOR-1 (Supplementary Fig. S1). Further, lentiviral over-expression of NOR-1 induced cIAP2 expression (mRNA and protein) in both human VSMC (Fig. 3a,b) and endothelial cells (Fig. 3c,d). The over-expression of NOR-1 in these cells was verified by real-time PCR and western blot (Fig. 3a–d).

### NOR-1 induces cIAP2 promoter activity through a NBRE

**Figure 4:**
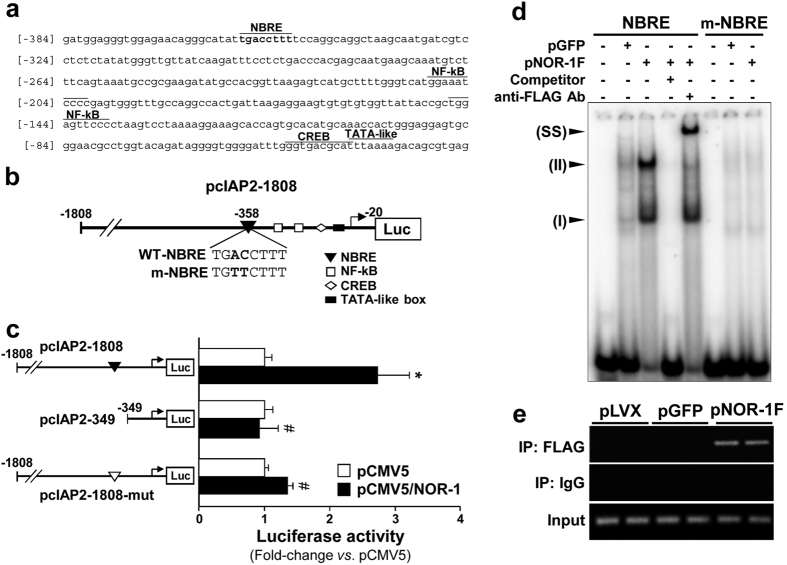
NOR-1 regulates cIAP2 promoter activity through a NBRE site. (**a**) cIAP2 proximal promoter sequence. *Cis*-acting regulatory elements and the putative NBRE are indicated. (**b**) Scheme showing the putative NBRE(−358/−351) site present in the cIAP2 promoter region cloned into the pGL3 reporter vector (pcIAP2-1808). The consensus NBRE site (WT-NBRE) is shown and mutated base pairs (m-NBRE) are indicated in bold. (**c**) Luciferase activity (normalized by Renilla) from cells co-transfected with a NOR-1 expression vector (pCMV5/NOR-1; black bars) or the corresponding empty plasmid (pCMV5; white bars) together with different pGL3/cIAP2 constructs. The activity of a construct mutated in the NBRE site (white triangle) is also shown (n = at least 6). *P < 0.0001 *vs*. cells co-transfected with pCMV5; ^#^P < 0.0001 *vs*. cells co-transfected with pCMV5/NOR-1 and pcIAP2-1808. (**d**) Representative autoradiograms of EMSA performed with a cIAP2 probe containing the NBRE(−358/−351) site (NBRE) and nuclear protein extracts from VSMC transduced with lentiviral vectors to express NOR-1-FLAG (pNOR-1F) or EGFP (pGFP). The position of the complexes up-regulated by NOR-1 (I and II) is indicated. Competition assays with a molar excess of unlabelled probe (100-fold; Competitor) and supershift assays with a specific antibody against the FLAG sequence (anti-FLAG Ab) were performed. EMSA carried out with a mutated NBRE probe (m-NBRE) is also shown. SS: supershifted complex. (**e**) Representative image of an agarose gel electrophoresis corresponding to a ChIP assay showing the relative *in vivo* association of NOR-1 with the human cIAP2 promoter in VSMC that over-expressed NOR-1-FLAG (pNOR-1F), GFP (pGFP) or pLVX vector (pLVX). Sheared chromatin was immunoprecipitated with an anti-FLAG antibody (IP:FLAG) or a non-specific IgG (IP:IgG). The enrichment of NOR-1 was assessed by PCR using cIAP2 promoter specific primers. Equal input DNA and control IgG inmunoprecipitations are shown.

The data described above suggest that NOR-1 modulates cIAP2 transcription. *In silico* analysis revealed the presence of a putative NBRE binding site in the proximal region of cIAP2 promoter (Fig. 4a). To analyze the functionality of this element, cells were transiently co-transfected with a pGL3 luciferase reporter plasmid harbouring approximately 1.8 Kbp of cIAP2 promoter (pcIAP2-1808) (Fig. 4b) and an expression vector for NOR-1 (pCMV5/NOR-1) or the empty vector (pCMV5). As shown in Fig. 4c, NOR-1 induced cIAP2 transcriptional activity about three-fold. NOR-1 responsiveness of cIAP2 promoter was lost in a deletion that excluded this putative NBRE site (−358/−351). Moreover, the mutation of this element by site-direct mutagenesis completely abrogated the NOR-1-dependent induction of cIAP2 promoter activity.

To further characterize the functionality of the NBRE(−358/−351) site, we performed electrophoretic mobility shift assays (EMSAs) using nuclear extracts from human VSMC transduced with a FLAG-tagged form of NOR-1. As shown in Fig. 4d, a differential pattern of retarded bands was observed in EMSAs carried out using nuclear extracts from pLVX/NOR-1-transduced cells respect those from control cells (transduced with pLVX/EGFP). NOR-1 over-expression increased the intensity of two of these complexes, that were competed by an excess of unlabelled probe, and one of them (complex II) was supershifted by an anti-FLAG antibody, indicating the binding of NOR-1 to this probe. Conversely, mutation of the NBRE probe abrogated the enhanced binding found in NOR-1-transduced cells. To confirm that NOR-1 actually binds to the cIAP2 promoter *in vivo*, Chromatin immunoprecipitation (ChIP) assays were performed in VSMC expressing FLAG-tagged NOR-1. NOR-1 directly bound to chromatin was immunoprecipitated using an anti-FLAG antibody. Conventional PCR was used to amplify a 129 bp genomic region (−393 to −265) surrounding the NBRE site of cIAP2 promoter. Consistent with data from EMSA, ChIP assays demonstrated that NOR-1 specifically binds to the cIAP2 promoter region encompassing the NBRE site (Fig. 4e). A control IgG did not precipitate detectable DNA and pre-immunoprecipitation samples evidenced equivalent DNA input. Therefore, NOR-1 specifically binds to this NBRE site in cIAP2 promoter.

In contrast to human cIAP2, mouse cIAP2 seems to be unresponsive to NOR-1, as far as no NOR-1 response elements could be detected in mouse cIAP2 promoter and regulation of cIAP2 by NOR-1 was neither observed in mouse aorta from NOR-1 transgenic mice (TgNOR-1) nor in VSMC from these animals (Supplementary Fig. S2a–c).Moreover, lentiviral over-expression of NOR-1 in mouse VSMC did not up-regulate cIAP2 (Supplementary Fig. S2d,e).

### NOR-1 mediated the up-regulation of cIAP2 induced by hypoxic stress

**Figure 5:**
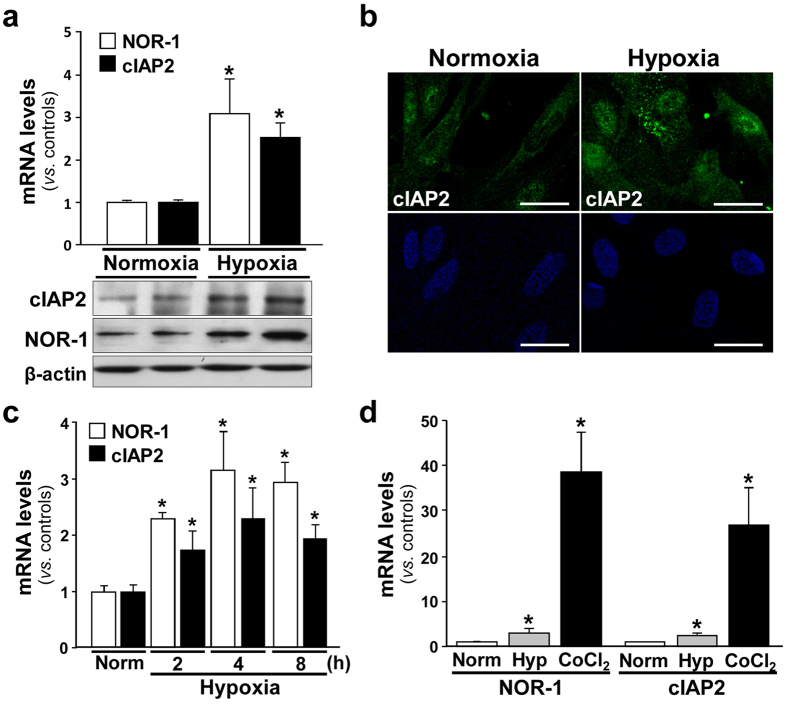
NOR-1 and cIAP2 are modulated by hypoxia in VSMC. (**a**) (upper panel) NOR-1 (white bars) and cIAP2 (black bars) mRNA levels analyzed by real-time PCR in VSMC exposed to hypoxia (0.2% O_2_) for 4 hours (n = at least 5), *P < 0.001 *vs*. cells maintained in normoxia. (lower panels) Representative western blot showing protein levels of NOR-1 and cIAP2 in VSMC exposed to hypoxia for 6 hours. Levels of β-actin are shown as a loading control. (**b**) Immunofluorescence confocal microscopy analysis showing cIAP2 (green), and nuclei (Hoechst stain, blue) in VSMC exposed to hypoxia for 6 hours. Bars: 25 μm. (**c**) NOR-1 (white bars) and cIAP2 (black bars) mRNA levels analyzed by real-time PCR in VSMC exposed to hypoxia for increasing times. (**d**) CoCl_2_ is a stronger inducer of NOR-1 and cIAP2 expression than hypoxia. mRNA levels of NOR-1 and cIAP2 analyzed by real-time PCR in VSMC exposed to hypoxia (Hyp) or CoCl_2_ (0.5 mM) for 4 hours (n = at least 6). *P < 0.0001 *vs*. cells maintained in normoxia (Norm).

**Figure 6:**
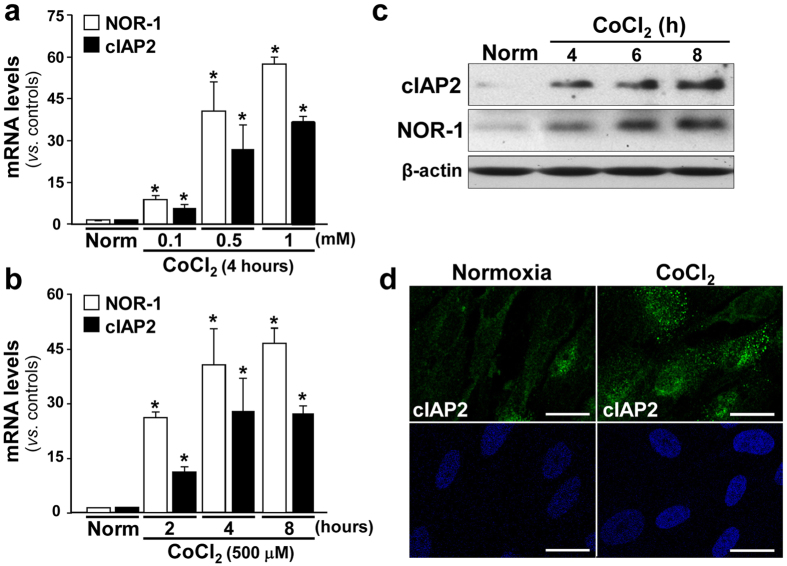
NOR-1 and cIAP2 are modulated by CoCl_2_ in VSMC in a dose- and time-dependent manner. (**a**,**b**) NOR-1 (white bars) and cIAP2 (black bars) mRNA levels analyzed by real-time PCR in VSMC exposed to increasing concentrations of CoCl_2_ (**a**) or CoCl_2_ (0.5 mM) for increasing times (**b**) (n = at least 5). *P < 0.0001 *vs*. cells maintained in normoxia (Norm). (**c**) Representative western blot showing protein levels of NOR-1 and cIAP2 in VSMC exposed to CoCl_2_ (0.5 mM) for increasing times. Levels of β-actin are shown as a loading control in western blot analysis. (**d**) Immunofluorescence confocal microscopy analysis showing cIAP2 (green) and nuclei (Hoechst stain, blue) in VSMC exposed to CoCl_2_ for 6 hours. Bar = 25 μm.

We have previously shown that hypoxia and, more potently, the hypoxia mimetic cobalt chloride (CoCl_2_), up-regulate NOR-1 in human endothelial cells^11^. In agreement with previous studies^30^ we detected an up-regulation of the hypoxia-inducible factor-1α (HIF-1α) in human AAA (Supplementary Fig. S3). In human VSMC, hypoxia induced both NOR-1 and cIAP2 (mRNA and protein levels) (Fig. 5a–c), and cobalt chloride was also a stronger inducer of both genes compared to hypoxia (Fig. 5d). Thus, we preferably used this compound to analyze the regulation of cIAP2 by NOR-1 in vascular cells exposed to hypoxic stress. Treatment of VSMC with CoCl_2_ resulted in similar time- and dose-dependent expression patterns of NOR-1 and cIAP2 (Fig. 6a,b). This parallelism was also observed in HUVEC (Supplementary Fig. S4), although in these cells both genes were less responsive than in VSMC. CoCl_2_ time-dependently increased NOR-1 and cIAP2 protein levels in VSMC as evidenced by Western blot (Fig. 6c) and confocal immunofluorescence (Fig. 6d).

**Figure 7:**
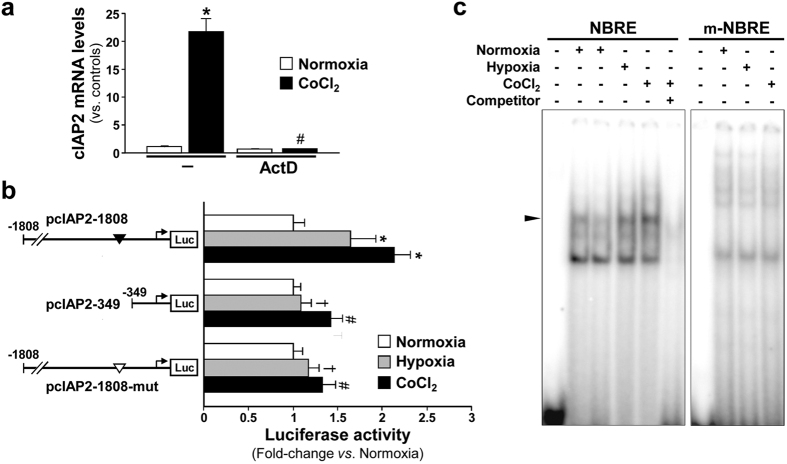
NOR-1 mediates the regulation of cIAP2 by hypoxia and CoCl_2_. (**a**) cIAP2 mRNA levels analyzed by real-time PCR in VSMC treated with CoCl_2_ (black bars) for 4 hours in the presence or absence of actinomycin D (ActD, 4 μM). Results are expressed relative to mRNA levels from VSMC maintained in normoxia and non-treated with ActD (controls) (n = 6). *P < 0.0001 *vs*. controls; ^#^P < 0.0001 *vs*. CoCl_2_ without ActD. 18S rRNA was used as endogenous control. (**b**) cIAP2 promoter activity in VSMC transfected with the constructs pcIAP2-1808, pcIAP2-349 or pcIAP2-1808-mut and maintained in normoxia (white bars), or exposed to hypoxia (grey bars) or to CoCl_2_ for 6 hours (black bars). Luciferase activity (normalized by Renilla) was measured and results are expressed relative to transcriptional activity in control VSMC (cells under normoxia conditions; white bars) (n = at least 8). *P < 0.0001 *vs*. cells maintained in normoxia; ^†^P < 0.001 *vs.* cells transfected with pcIAP2-1808 and exposed to hypoxia; ^#^P < 0.001 *vs.* cells transfected with pcIAP2-1808 and exposed to CoCl_2_. (**c**) Representative autoradiograms of EMSA performed with a cIAP2 probe containing the NBRE(−358/−351) site (NBRE) and nuclear protein extracts from VSMC maintained under normoxia, or exposed to hypoxia or CoCl_2_ for 4 hours. The position of the complex up-regulated by hypoxia and CoCl_2_ (arrowhead) is indicated. Competition assays with a molar excess of unlabelled probe (100-fold; Competitor) were performed. EMSA carried out with a mutated NBRE probe (m-NBRE) is also shown.

NOR-1, which has a functional hypoxia response element in its promoter^11^, is up-regulated by hypoxia in a HIF-1α-dependent manner. Therefore, as expected, HIF-1α silencing prevented the induction of NOR-1 in response to the hypoxia mimetic CoCl_2_, and consequently also abrogated the increase of cIAP2 mRNA levels (Supplementary Fig. S5). Finally, we analyzed whether NOR-1 was directly responsible for the up-regulation of cIAP2 by hypoxia and CoCl_2_. Actinomycin D abrogated the CoCl_2_-induced cIAP2 up-regulation (Fig. 7a) suggesting the involvement of a transcriptional mechanism. To assess the contribution of NOR-1, VSMC were transiently transfected with the pcIAP2-1808 construct and exposed to hypoxia or CoCl_2._ The increase in the transcriptional activity of this construct mediated by hypoxia or CoCl_2_ was significantly reduced when the NBRE (−358/−351) site was deleted or mutated (Fig. 7b). Finally, hypoxia and more evidently CoCl_2_ increased the binding to the NBRE (−358/−351) site in EMSA (Fig. 7c). These data indicate that NOR-1 is directly involved in the modulation of cIAP2 by hypoxic stress.

### The NOR-1 anti-apoptotic effect is mediated by cIAP2

**Figure 8:**
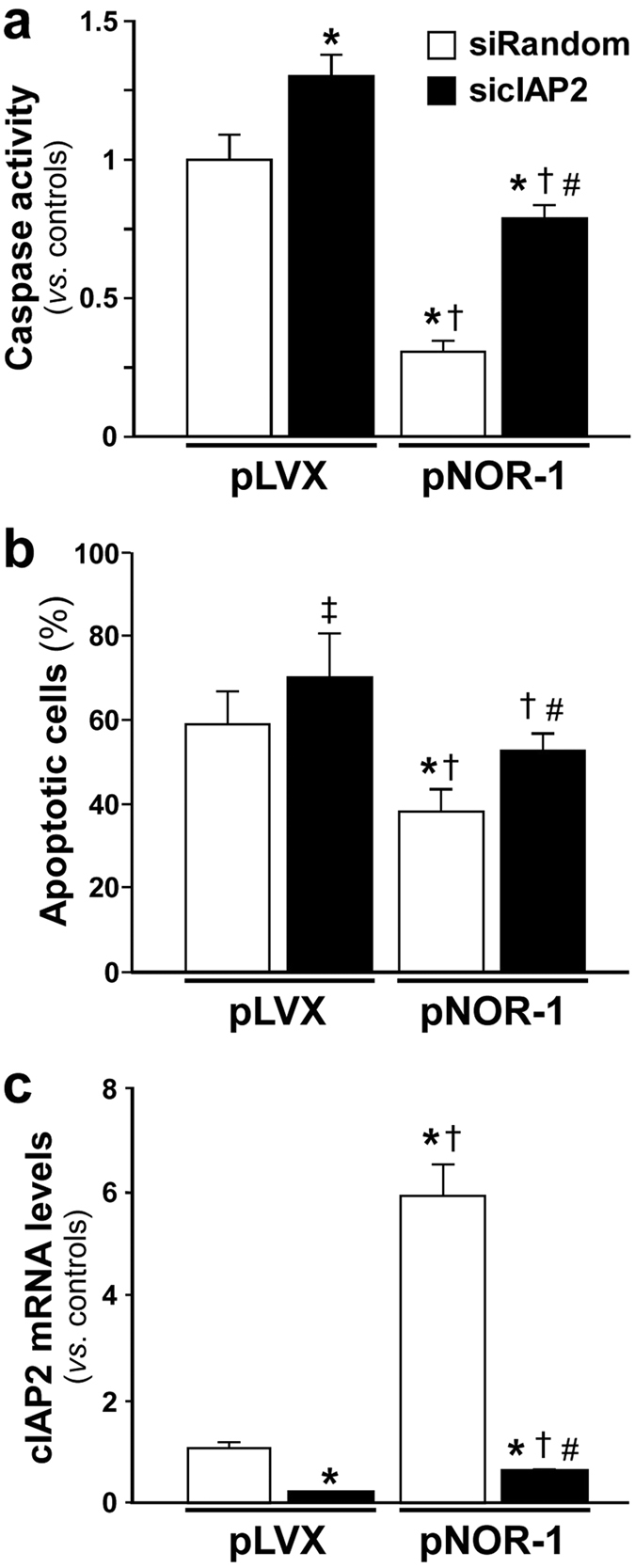
cIAP2 is involved in the pro-survival effect of NOR-1 in VSMC exposed to CoCl_2_. VSMC were lentiviral transduced to over-express NOR-1 (pNOR-1) or transduced with the pLVX empty vector (pLVX), transfected with a siRNA against cIAP2 (sicIAP2; black bars) or a random siRNA (siRandom; white bars), and exposed to CoCl_2_ for 16 hours. (**a**) Analysis of caspase 3/7 activity in VSMC treated as indicated above. Results are relative to caspase activity in VSMC control (pLVX transduced cells transfected with siRandom) (n = 7). *P < 0.001 *vs*. pLVX and siRandom; ^†^P < 0.0001 *vs*. pLVX and sicIAP2; ^#^P < 0.0001 *vs*. pNOR-1 and siRandom. (**b**) Apoptosis evaluated by FACS after annexin V-PC5 staining in VSMC treated as indicated above. Data were expressed as percentages of the total cell population (n = 6). *P < 0.0001 *vs*. pLVX and siRandom; ^†^P = 0.016 *vs*. pLVX and siRandom; ^†^P < 0.001 *vs*. pLVX and sicIAP2; ^#^P = 0.004 *vs*. pNOR-1 and siRandom. (**c**) mRNA levels of cIAP2 analyzed by real-time PCR in cells as indicated above. Results are expressed relative to cIAP2 mRNA levels in control cells (VSMC transduced with pLVX and transfected with siRandom) (n = 6). *P < 0.005 *vs*. pLVX and siRandom; ^†^P < 0.05 *vs*. pLVX and sicIAP2; ^#^P < 0.0001 *vs*. pNOR-1 and siRandom.

We have previously reported that NOR-1 is a pro-survival transcription factor for endothelial cells exposed to hypoxic stress^11^. In agreement with this, the rate of apoptosis (estimated either as the level of caspase-3/7 activity or analyzed by fluorescence-activated cell sorting [FACS]) in VSMC exposed to hypoxic stress (0.5 mM CoCl_2_) was lower in cells over-expressing NOR-1, and this effect was partially abrogated when cIAP2 was silenced (Fig. 8a,b). The efficiency of cIAP2 silencing in these experimental conditions was assessed by real-time PCR (Fig. 8c).

## Discussion

NR4A receptors have been implicated in a variety of cellular processes including the transduction of hormonal, inflammatory, mitogenic, apoptotic and differentiative signals. Interestingly, while in some cell types such as myeloid and T cells they seem to play a key pro-apoptotic role^31^,^32^,^33^,^34^ in other cells and tissues they are pro-survival factors and protect against apoptosis induced by stressful stimuli^35^,^36^,^37^,^38^. We and others have recently involved NOR-1 in vascular remodelling^14^,^15^,^16^,^17^,^18^. NOR-1 regulates vascular cell spreading, migration and proliferation^11^,^14^,^17^,^26^,^28^,^29^, and is implicated in the survival response of vascular cells to hypoxic stress^11^. In this work, we report that cIAP2, a gene encoding a well-known anti-apoptotic protein, is a downstream target of NOR-1 that mediates NOR-1 pro-survival effects in vascular cells exposed to hypoxic stress. Furthermore, we show that the expression levels of NOR-1 and cIAP2 are increased and positively correlated in AAA, a pathological condition in which vascular cells are forced to adapt to stress conditions that compromise cell survival.

We have previously suggested that cIAP2 could be a target of NOR-1^11^. Interestingly, we observed that both NOR-1 and cIAP2 were up-regulated and colocalized in VSMC in human AAA. To gain more insight into the regulation of cIAP2 by NOR-1 in vascular cells we used both loss- and gain-of-function approaches. NOR-1 knockdown experiments in human VSMC confirmed our previous results from similar approaches in human endothelial cells showing that cIAP2 expression depends on NOR-1^11^. What is more, lentiviral over-expression of NOR-1 significantly up-regulated cIAP2 in both human VSMC and endothelial cells. Finally, in transient co-transfection assays using a luciferase reporter plasmid (harbouring the proximal promoter sequence of cIAP2) and a NOR-1 expression vector we show that this nuclear receptor is able to regulate cIAP2 transcriptional activity. Indeed, promoter deletion and site-directed mutagenesis analysis identified a fully conserved NBRE motif (NBRE −358/−351) critically involved in the NOR-1-mediated induction of cIAP2 promoter activity. The direct binding of NOR-1 to this site was demonstrated *in vitro* and *in vivo* by EMSA and ChIP assays respectively. Few transcription factors have been characterized in the control of cIAP2 expression, among them NFκB, CREB or STAT3 39,40. Therefore, our results characterizing a functional NBRE site in cIAP2 promoter and involving NOR-1 in the regulation cIAP2 expand the knowledge on the cIAP2 promoter structure and regulation.

Under pathological conditions such as AAA, hypoxia is a powerful trigger of vascular remodelling^30^,^41^. cIAP2 expression is induced by a variety of stimuli including hypoxia; however, no functional hypoxia responsive elements (HREs) in cIAP2 promoter have been characterized and the mechanism by which hypoxia mediates the up-regulation of cIAP2 is unclear^11^,^42^,^43^,^44^. The transcriptional response of mammalian cells to hypoxia is largely mediated by HIF-1^45^,^46^, but additional transcription factors cooperate in the complex response of cells to hypoxia in a HIF-dependent or independent manner^47^. Nowadays, this network of transcription factors that regulate structural genes involved in the adaptation of vascular cells to hypoxic stress is not completely understood. In the present work, we show that hypoxia and more potently a hypoxia mimetic (CoCl_2_) concomitantly up-regulate NOR-1 and cIAP2 in a time- and dose-dependent manner. CoCl_2_ was a stronger stimulus stabilizing HIF-1α, the transcription factor responsible for the up-regulation of NOR-1 by hypoxic stress^11^, than physical hypoxia (Supplementary Fig. S6) resulting in greater induction of both NOR-1 and cIAP2. Moreover, we demonstrate that the NBRE(−358/−351) site is involved in the increase of cIAP2 transcriptional activity triggered by hypoxia. We have previously linked NOR-1 to the survival response of cells to hypoxic stress^11^. Our present results in VSMC exposed to CoCl_2_ show that while NOR-1 over-expression limits cell apoptosis associated to high cIAP2 expression levels, cIAP2 silencing partially counteracts this effect. Concerning the potential biological significance of this in AAA, although in cell culture physical hypoxia was a discrete modulator of NOR-1/cIAP2 and a poor inducer of human VSMC apoptosis, it should be taken into account that not only hypoxia but also inflammation, a well known inducer of NOR-1 and a trigger of apoptosis^48^,^49^, may work in concert producing additive or synergistic effects on gene expression and cell apoptosis. Therefore, the induction of cIAP2 mediated by NOR-1 seems to be responsible, at least in a part, of the pro-survival effect of NOR-1 in cells exposed to hypoxic stress and probably in more complex pathological settings.

In summary, these results expand to VSMC our previous observations identifying NOR-1 as a pro-survival factor for human endothelial cells, evidence that NOR-1 regulates the expression of cIAP2 through a transcriptional mechanism, and show that this anti-apoptotic protein is responsible of the survival response dependent on NOR-1. It should be noted, however, that our *in silico* analysis identified the NBRE responsive for NOR-1 regulation in the human cIAP2 promoter but not in mouse cIAP2 promoter, and our experimental results discard cIAP2 as a NOR-1 target gene in this species. Thus, unfortunately further testing on the functional significance of cIAP2 modulation by NOR-1 using small animal models is not possible. Previous studies from us and others indicate that there are notable differences between human and murine rodents in both the mechanisms that regulate NOR-1^50^ and NOR-1-targeted genes^51^,^52^. Consequently, before extrapolating biomedical consequences from NOR-1 biology in mouse models, it is mandatory to corroborate the data in humans. Further studies are needed to determine the relative contribution of the different transcription factors and structural genes responsive to hypoxia to depict the complex network of genes/proteins involved in the adaptive response of mammalian cells to hypoxic stress.

## Materials and Methods

### Human tissue samples

AAA samples were obtained from patients who underwent open repair surgery for AAA at Hospital de la Santa Creu i Sant Pau (HSCSP). Normal abdominal aortas were obtained from multiorgan donors. Specimens for immunohistochemical studies were fixed overnight in 4% paraformaldehyde/0.1 M PBS (pH 7.4), embedded in paraffin and sectioned into 5 μm sections. Specimens for Western blot and PCR analysis were snap-frozen in liquid nitrogen and stored at −80 °C until processed^53^. The study was approved by the HSCSP Ethics Committee (13/103/1491) and was conducted according to the Declaration of Helsinki. Written informed consent was obtained from each patient.

### Animals

Gene expression was analyzed in aorta from mice that over-expresses NOR-1 in VSMC^18^ and wild-type control littermates. Mice were bred in the Animal Experimentation Unit (CSIC-ICCC). Animals were euthanized under ketamine (75 mg/kg)/medetomidine (1 mg/kg) anaesthesia, and aorta was excised, frozen in liquid nitrogen and stored at −80 °C. All procedures were reviewed and approved by the Ethical Committee at the Centro de Investigación Cardiovascular (Barcelona, Spain; ICCC-055) as stated in Law 5/1995 (Generalitat de Catalunya) and follow the Spanish Policy for Animal Protection RD53/2013, which meets the European Union Directive 2010/63/UE.

### Cell cultures

VSMC were obtained from human non-atherosclerotic arteries of hearts removed in transplant surgeries at the HSCSP as described^53^. All the procedures were approved by the Reviewer Institutional Committee on Human Research of the HSCSP (12/007/1292) and conform to the Declaration of Helsinki and written informed consent was obtained from each patient. VSMC and HUVEC (Lonza) were cultured as described^54^. Cells were stimulated with CoCl_2_ or exposed to hypoxia (0.2% O_2_). When needed, the cells were pre-treated with actinomycin D. VSMCs from mouse aorta were obtained by the explant technique^18^. Mouse VSMC and HEK293T and HeLa cell lines were cultured in DMEM supplemented with 10% FCS, 2 mM L-glutamine and antibiotics.

### Generation of lentiviral particles and transduction

The human NOR-1 cDNA was obtained from a pBlueScript-NOR1 construct, kindly provided by Dr. Ohkura (National Cancer Center Research Institute, Japan)^55^, and the mouse NOR-1 cDNA linked to a FLAG sequence was obtained from the plasmid pCMV5/NOR-1-FLAG, kindly provided by Dr. Hastie (University of Dundee)^56^. The pLVX/NOR-1, pLVX/NOR-1-FLAG and pLVX/EGFP were obtained as described^48^. These constructs and the empty pLVX-Puro vector (pLVX) were transfected in HEK293T cells according to Lenti-X^TM^ Lentiviral Expression System Kit (Clontech). After 48 h, supernatants containing viral particles were harvested and titrated. Lentiviral transductions of VSMC and HUVEC were performed for 48 h at a multiplicity of infection of 15 in presence of polybrene (8 μg/mL). Transduced cell populations were enriched by puromycin selection. pLVX and pLVX/EGFP vectors were used as controls obtaining similar results.

### Transfections with small interfering RNA (siRNA)

siRNAs against NOR-1 (Silencer Select Pre-designed siRNA s15541 [Ambion] and ON-TARGET plus SMARTpool L-003428-00-0005 [Dharmacon]), cIAP2 (ON-TARGET plus SMARTpool L-004099-00-0005) and HIF-1α (ON-TARGET plus SMARTpool L-004018-00-0005) were used. The Silencer Select Negative Control #1 was used as a control. Lipofectamine^TM^ RNAiMAX (Invitrogen) was used for siRNA delivery^57^. Cells were transfected with 20–50 nM siRNA, using 7.5 μL of Lipofectamine RNAiMAX Reagent. After transfection (8–24 h), the medium was replaced and cells were incubated for at least 16 h. In some experiments, VSMC were serum-deprived for 24–48 h and exposed to CoCl_2_.

### Gene expression: real-time PCR

Total RNA was extracted using TRIsure^TM^ (Bioline) and reverse-transcribed with the High Capacity cDNA Reverse Transcription Kit (Applied Biosystems). mRNA levels were assessed by real-time PCR using TaqMan^TM^ gene expression assays-on-demand (Applied Biosystems). TATA-binding protein (TBP) was used as endogenous control (unless otherwise stated)^18^.

### Generation of cIAP2 promoter constructs

A 1.8 kb fragment corresponding to nucleotides −1808 to −20 of the human cIAP2 promoter^58^ was generated by PCR and cloned into the pGL3 vector (pcIAP2-1808). The primers used were: 5′-CGTGCGGTACCACACTTGGCTCATTTTTGT-3′ (forward; *Kpn*I site is underlined) and 5′-GGAGGGCTCGAGTCTCACGCTGTCTTTTAA-3′ (reverse; *Xho*I site is underlined). The PCR product was digested with *Kpn*I and *Xho*I and cloned into the pGL3 vector. A promoter deletion construct (−349 to −20; pcIAP2-349) was generated using the reverse primer indicated above and the following forward primer: 5′-TGACCGGTACCAGGCAGGCTAAGCAATGA-3′ (*Kpn*I site is underlined). The putative NBRE(−358/−351) site located in cIAP2 promoter was mutated using the QuikChange^TM^ Site-Directed Mutagenesis Kit (Stratagene) (pcIAP2-1808-mut) and primers 5′-TGGAGAACAGGGCATATTGttCTTTTCCAGGCAGGCTAAG-3′ and 5′-CTTAGCCTGCCTGGAAAAGaaCAATATGCCCTGTTCTCCA-3′ (NBRE site is underlined and changes are indicated in lower case letters).

### Transient transfection and Luciferase assays

VSMC were transfected using Lipofectamine LTX^TM^ and Plus Reagent (Invitrogen). cIAP2 constructs were co-transfected together with pCMV5/NOR-1 expression plasmid or the corresponding empty vector (pCMV5)^52^. Luciferase activity was determined in cell lysates using the Dual-Luciferase^TM^ Reporter Assay System (Promega). Results were expressed as the ratio of firefly to renilla activity.

### EMSA

EMSA was performed using 5 μg of nuclear extracts obtained from human VSMC^59^. Double-stranded DNA probes containing the putative wild-type NBRE(−358/−351) site in cIAP2 promoter (5′-GGGCATATTGACCTTTTCCAGGCA-3′) and its mutated form (5′-GGGCATATTGttCTTTTCCAGGCA-3′) were used. DNA probes were labelled with [γ-^32^P]-ATP using T4 polynucleotide kinase and purified on a Sephadex G-50 column. In competition assays, the unlabelled probe was added before the labelled one and was incubated for 10 min. For supershift assays, nuclear extracts were pre-incubated for 25 min with 2 μg of an anti-FLAG antibody (F1804, Sigma-Aldrich). Protein-DNA complexes were resolved by electrophoresis on 5% polyacrylamide gels in 0.3X TBE. Gels were dried and subjected to autoradiography using a Storage Phosphor Screen (GE Healthcare). Shifted bands were detected using a Typhoon 9400 Scanner (GE Healthcare).

### ChIP assay

VSMC were cross-linked with 1% formaldehyde for 10 min. The cross-link reaction was stopped by adding glycine (100 mM). Nuclei were isolated as described^52^. Chromatin was sheared by sonication and an aliquot was saved and stored as input DNA. Supernatants were then immunoprecipitated with 5 μg of anti-FLAG antibody (F1804) or an IgG as a control. Immune complexes were recovered by addition of A/G-Agarose beads. After washing, complexes were extracted, cross-link was reversed and the DNA was purified and concentrated. Purified DNA was analyzed by PCR amplifying a 129 bp DNA fragment (forward primer: 5′- TGTATGGCGGATGGAGGGTGGA-3′; reverse primer: 5′-AGACATTTGCTTCATTGCTCG-3′). The amplified PCR products were run by electrophoresis on ethidium bromide stained agarose gels^60^.

### Caspase activity assay

Caspase activity was determined in VSMC transduced with the pLVX/NOR-1 construct or the pLVX vector and transfected with a pool of siRNAs against cIAP2 or the corresponding siRandom. After transfection, cells were serum deprived for 48 h and stimulated with CoCl_2_ 16 h. Then, cells were lysed and caspase activity was measured in cell lysates using the Caspase-Glo^TM^ 3/7 assay Kit (Promega) and a luminometer. Data were normalized by protein content.

### Analysis of apoptosis by FACS

VSMC were processed as indicated in the previous section. Cells were trypsinized, pooled with cells present in the cell supernatants, resuspended in binding buffer, and incubated with annexin V conjugated with fluorescein isothiocyanate (FITC) and propidium iodide (FITC Annexin V Apoptosis Detection Kit I, BD Pharmigen) according to the manufacturer^11^. Annexin V-PC5 and PI binding was analyzed by FACS (Epics XL flow cytometer; Beckman Coulter). Data were gated for viable cells (annexin V− and PI−), damaged cells (annexin V− and PI+), apoptotic cells (annexin V+ and PI−) and late apoptotic cells (annexin V+ and PI+). Data were expressed as percentages of the total cell population.

### Western blot analysis

Cellular and tissue extracts were obtained as described^53^. Lysates were resolved by SDS-PAGE under reducing conditions and electrotransfered onto Immobilon polyvinylidene difluoride membranes. Membranes were probed using antibodies against NOR-1 (H00008013-M06, Abnova), cIAP2 (ab32059, Abcam), HIF-1α (NB-100-449, Novus Biologicals) and β-actin (A5441, Sigma-Aldrich), followed by appropriate horseradish peroxidase-conjugated secondary antibodies and a chemiluminescent detection system. Equal loading was verified by Ponceau staining and by β-actin levels.

### Immunocytochemistry

Cells were fixed in ice-cold 4% paraformaldehyde, blocked and incubated with an anti-cIAP2 antibody (ab32059) in PBS containing 5% BSA, overnight at 4 °C. An Alexa fluor 488 goat anti-rabbit immunoglobulin (Molecular Probes) was used as secondary antibody. For nuclei, Hoechst 33342 trihydrochloride trihydrate (H3570, Invitrogen) was used. Controls without the primary antibody were included in all procedures. Cells were mounted with ProLong^TM^ Mounting Medium (Molecular Probes) and analyzed by confocal microscopy.

### Immunostaining of human arteries

Consecutive deparaffinized sections were rehydrated, subjected to antigen retrieval in 10 mM citrate buffer pH 6.0 (95 °C for 20 min) and blocked for 30 min with 10% goat or horse serum/PBS. Antibodies against cIAP2 (ab32059), NOR-1 (H00008013-M06) and HIF-1α (NB-100-449) were used. Sections were incubated with the corresponding biotinylated secondary antibodies. Immunocomplexes were detected after incubation with Vectastain Elite ABC reagent (PK6100, Vector) and DAB substrate.

### Statistical analysis

Data are expressed as mean ± s.d. (unless otherwise state). Significant differences were established by Student’s t-test or one-way ANOVA, according to the number of groups compared, using the GraphPad Instat program (GraphPad Software V2.03) (GraphPad Software Inc.). When normality failed we used the Mann-Whitney rank sum test to compare two groups. To determine association between variables a Spearman Rank Order Correlation analysis was performed with SigmaPlot software V11.0 (Systat Software Inc.). Differences were considered significant at P < 0.05.

## Additional Information

**How to cite this article**: Alonso, J. *et al*. NOR-1/NR4A3 regulates the cellular inhibitor of apoptosis 2 (cIAP2) in vascular cells: role in the survival response to hypoxic stress. *Sci. Rep.* **6**, 34056; doi: 10.1038/srep34056 (2016).

## Supplementary Material

Supplementary Information
