# Supplementary material for: NOR-1/NR4A3 regulates the cellular inhibitor of apoptosis 2 (cIAP2) in vascular cells: role in the survival response to hypoxic stress
Source: Sci Rep. 2016 Sep 22;6:34056. doi: 10.1038/srep34056 (PMC5032021; doi:10.1038/srep34056)
Supplement: Supplementary Information [file srep34056-s1.pdf]

Supplementary Information

NOR-1/NR4A3 regulates the cellular inhibitor of apoptosis 2 (cIAP2) in vascular cells: role in the survival response to hypoxic stress

Judith Alonso[1], María Galán[1,2], Ingrid Martí-Pàmies[1], José María Romero[2], Mercedes Camacho[2], Cristina Rodríguez[1] & José Martínez-González[1*]

[1]Centro de Investigación Cardiovascular (CSIC-ICCC), Instituto de Investigación Biomédica Sant Pau (IIB-Sant Pau), c/Sant Antoni Maria Claret 167, 08025 Barcelona, Spain.
[2]Laboratorio de Angiología, Biología Vascular e Inflamación y Servicio de Cirugía Vascular, IIB-Sant Pau, c/Sant Antoni Maria Claret 167, 08025 Barcelona, Spain.

*Corresponding author: José Martínez-González, Centro de Investigación Cardiovascular (CSIC-ICCC), Hospital de la Santa Creu i Sant Pau, Avda. Sant Antoni Maria Claret 167. 08025 Barcelona. Spain. TEL: +34 935565896; FAX: +34 935565559; E-mail: jmartinez@csic-iccc.org.

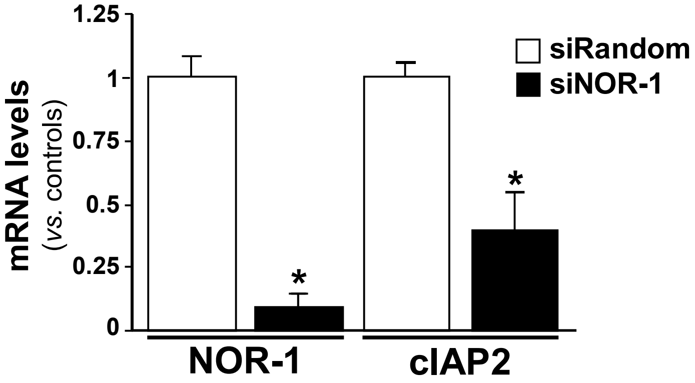

Supplementary Fig. S1. NOR-1 knockdown down-regulates cIAP2 expression in VSMC. Human VSMC were transfected with a pool of siRNAs against NOR-1 (siNOR-1; ON-TARGET plus SMARTpool L-003428-00-0005; black bars) or a control random siRNA (siRandom; white bars) and mRNA levels of NOR-1 and cIAP2 were analyzed by real-time PCR (n = 6). *, P < 0.0001 *vs.* siRandom.

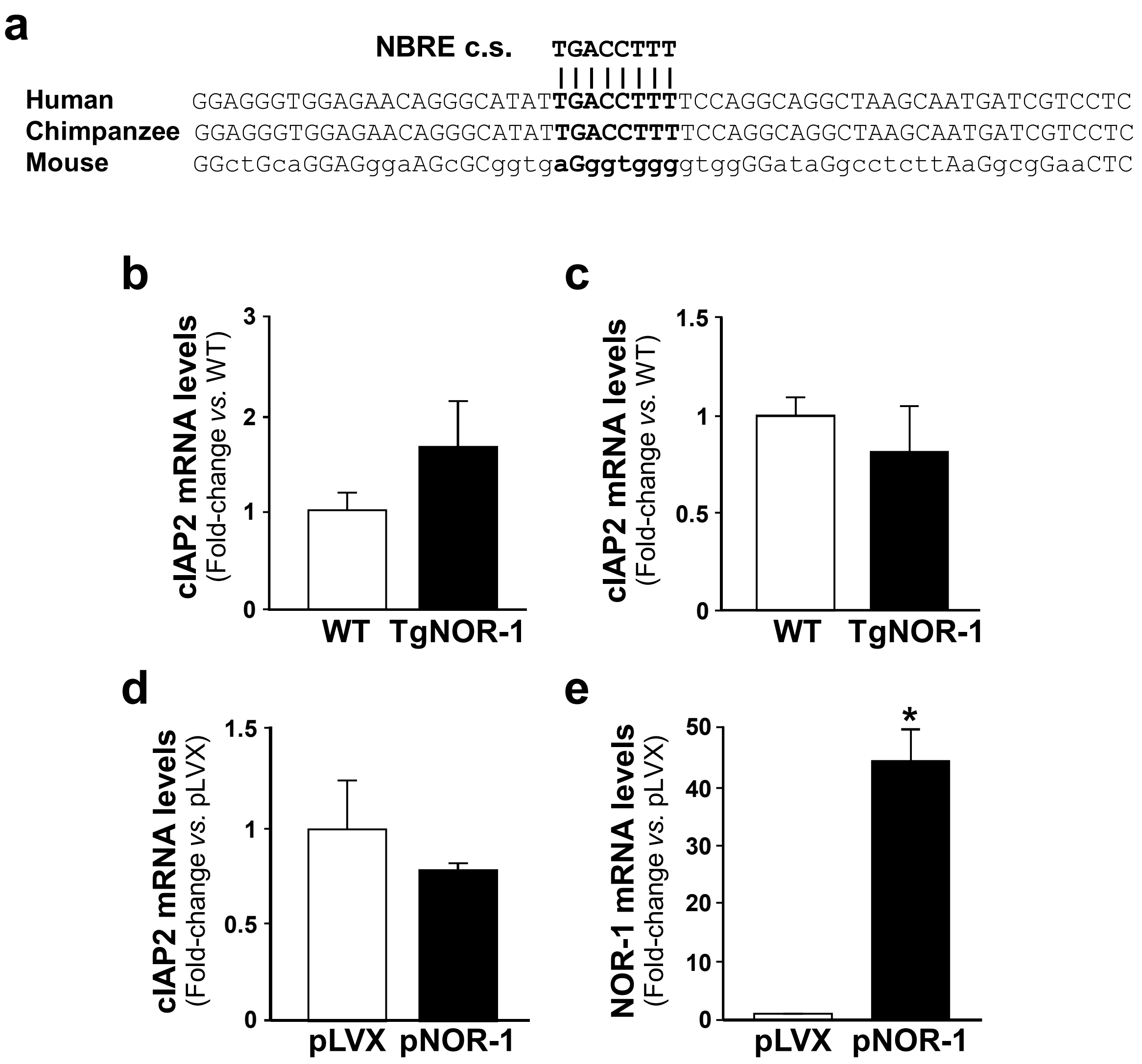

Supplementary Fig. S2. cIAP2 is not regulated by NOR-1 in mouse. (**a**) Alignment of the proximal region corresponding to the cIAP2 promoter from different species including human and mouse. Non-conserved positions are indicated in lower case. The NBRE consensus sequence (NBRE c.s.) is shown at the top. (**b** to **e**) cIAP2 was not regulated by NOR-1 over-expression in mice. cIAP2 mRNA levels were analyzed by real-time PCR in mouse aorta from wild-type (WT; n= 6) and transgenic mice that specifically over-express human NOR-1 in VSMC (TgNOR-1; n= 6) (**b**), in cultures of VSMC from these animals (**c**), and in mouse VSMC transduced to over-express NOR-1 (**d**). (**e**) NOR-1 mRNA levels in mouse VSMC transduced with pLVX-NOR-1 (containing the NOR-1 cDNA from mouse) or pLVX. Data are expressed as mean ± s.e.m (**b**) and mean ± s.d (**c** to **e**) (n = at least 5). *P < 0.0001 *vs*. pLVX.

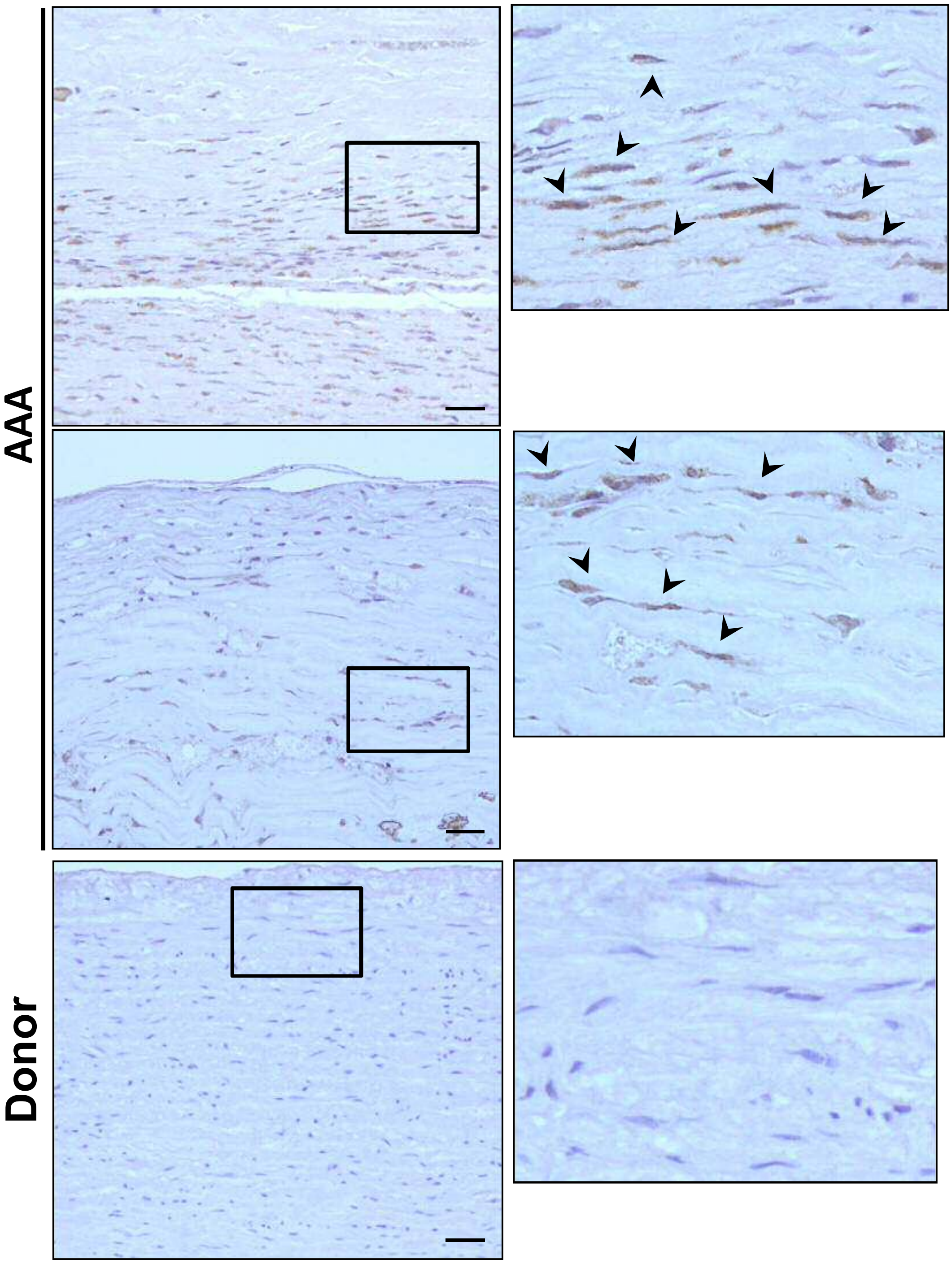

Supplementary Fig. S3. Hypoxia-inducible factor-1α (HIF-1α) is up-regulated in human abdominal aortic aneurysm (AAA). Representative images showing the immunostaining for HIF-1α in elongated nuclei from VSMC in media of aorta sections from human AAA (upper panels). Representative images showing negative immunostaining for HIF-1α in aorta sections from healthy donors are shown (lover panels). Bar = 50 μm.

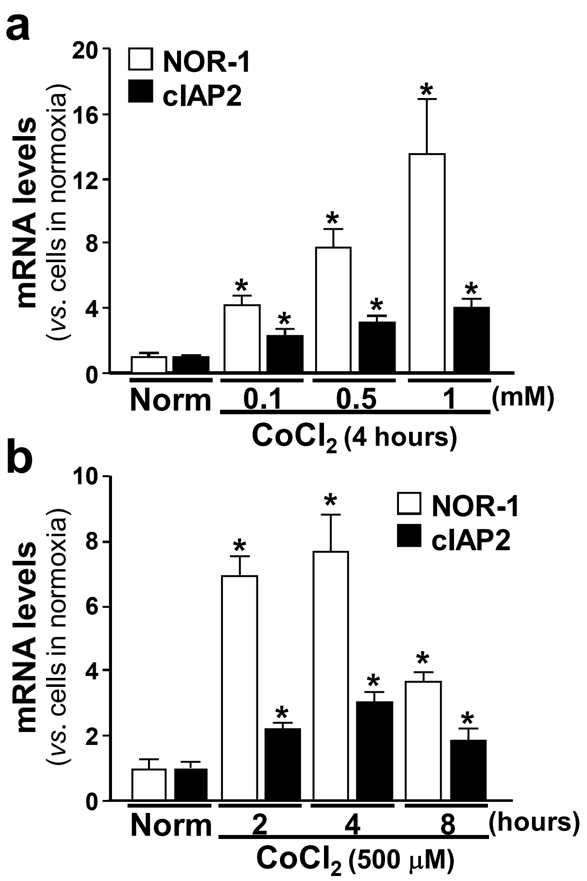

Supplementary Fig. S4. In HUVEC NOR-1 and cIAP2 are induced by $CoCl_2$ in a dose- and time-dependent manner. Analysis of mRNA levels of NOR-1 (white bars) and cIAP2 (black bars) by real-time PCR in HUVEC exposed to increasing concentrations of $CoCl_2$ (**a**) or $CoCl_2$ (500 μM) for increasing times (**b**) (n = at least 5), *P < 0.0005 *vs.* cells exposed to normoxia (Norm).

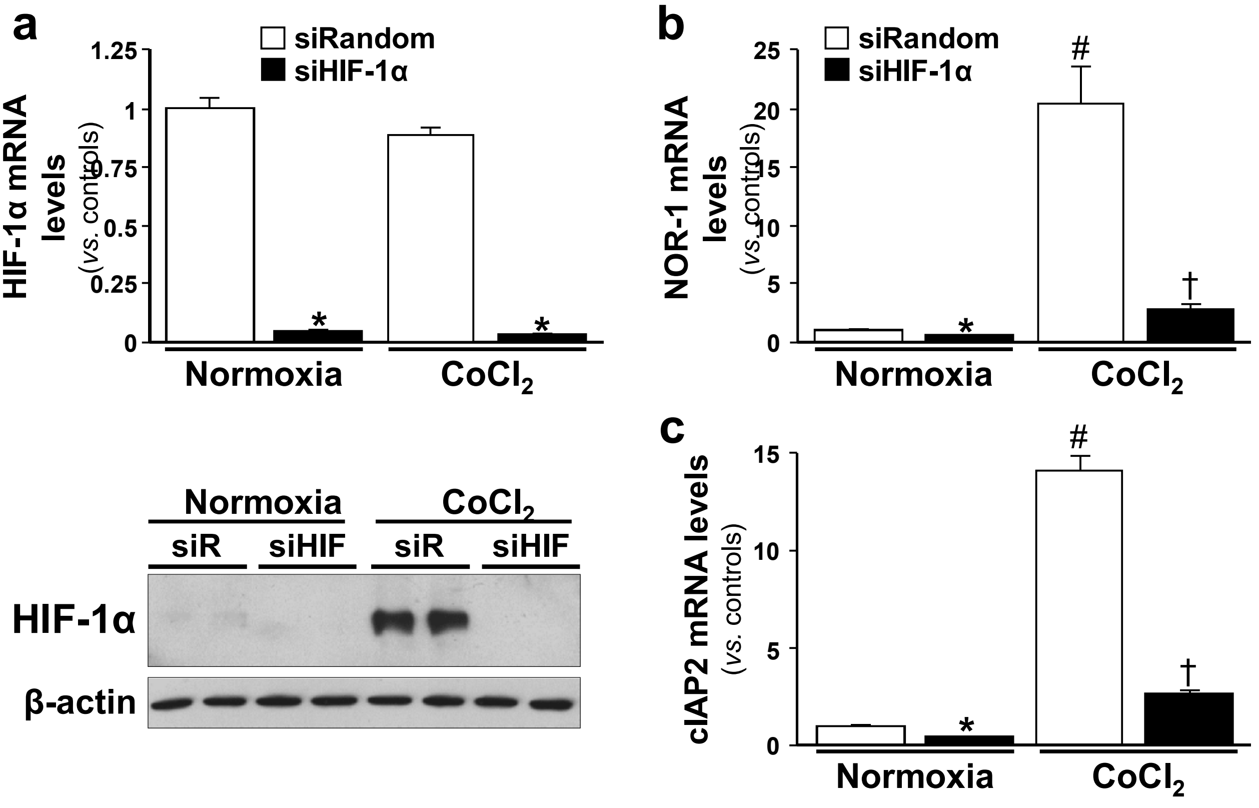

Supplementary Fig. S5. HIF-1α silencing prevents the up-regulation of NOR-1 and cIAP2 induced by the hypoxia mimetic $CoCl_2$. (**a**) mRNA (upper panel) and protein levels (lower panel) of HIF-1α from VSMC transfected with a pool of siRNA against HIF-1α (siHIF-1α, siHIF) or with control siRNA (siRandom, siR) and maintained under normoxia or treated with the hypoxia mimetic $CoCl_2$ (0.5 mM for 4 h). Upper panel: (n = 6); *, $P < 0.0001$ *vs*. siRandom with the same treatment. Levels of β-actin are shown as a loading control in western blot analysis. (**b**) mRNA levels of NOR-1 analyzed by real-time PCR from VSMC treated as indicated in (a) (n = 6). *, $P < 0.05$ *vs*. siRandom under normoxia; #, $P < 0.0001$ *vs*. siRandom under normoxia; †, $P < 0.0001$ *vs.* siRandom treated with $CoCl_2$. (**c**) mRNA levels of cIAP2 analyzed by real-time PCR from VSMC treated as indicated in (a) (n = 6). *, $P < 0.01$ *vs*. siRandom under normoxia; #, $P < 0.0001$ *vs*. siRandom under normoxia; †, $P < 0.0001$ *vs*. siRandom treated with $CoCl_2$.

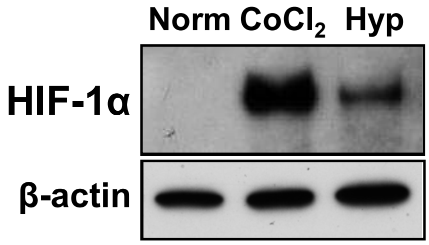

Supplementary Fig. S6. In human aortic VSMC, the hypoxia mimetic $CoCl_2$ triggers a stronger HIF-1α induction than physical hypoxia. HIF-1α protein levels from VSMC maintained under normoxia (Norm), treated with the hypoxia mimetic $CoCl_2$ (0.5 mM for 4 h) or exposed to hypoxia (Hyp; 0.2% O_2 for 4 h). Levels of β-actin are shown as a loading control in western blot analysis (n = 4).
